# Frequency Dependence of the Entanglement Entropy Production in a System of Coupled Driven Nonlinear Oscillators

**DOI:** 10.3390/e21090889

**Published:** 2019-09-13

**Authors:** Shi-Hui Zhang, Zhan-Yuan Yan

**Affiliations:** Department of Mathematics and Physics, North China Electric Power University, Baoding 071003, China; physxzhang@ncepu.edu.cn

**Keywords:** entropy production, driven nonlinear systems, driving frequency

## Abstract

Driven nonlinear systems have attracted great interest owing to their applications in quantum technologies such as quantum information. In quantum information, entanglement is a vital resource and can be measured by entropy in bipartite systems. In this paper, we carry out an investigation to study the impact of driving frequency on the entanglement with a bipartite system of two coupled driven nonlinear oscillators. It is numerically found that the time evolution of the entanglement entropy between the subsystems significantly depends on the driving frequency. The dependence curve of the entropy production on the driving frequency exhibits a pronounced peak. This means the entanglement between the subsystems can be greatly increased by tuning the driving frequency. Further analyses show that the enhancement of the entropy production by the driving frequency is closely related to the energy levels involved in the quantum evolution. This is confirmed by the results related to the quantum spectrum and the dispersion of the wave function in the phase space. Our work gives a convenient way to enhance the entanglement in driven nonlinear systems and throws light on the role of driven nonlinear systems in quantum information technologies.

## 1. Introduction

As is widely known, quantum entanglement is a remarkable feature of quantum mechanics and plays a central role in quantum information and communication theory [1,2]. It, in nature, is a type of quantum correlation, which is an essential ingredient for the quantum technologies. During recent years, quantum correlation that includes entanglement has attracted much attention [1,2,3,4,5,6,7]. Especially, great interest has been focused in the last decades on achieving, enhancing, and controlling quantum correlation, as quantum correlation is a key resource in quantum technologies [2,3,4,5]. For instance, a useful scheme is presented for generating high amount of bipartite entanglement by means of the beam splitter [3]. In addition, entanglement is investigated in nonlinear cavity QED, and its dynamical behavior is found to be very sensitive to the initial state and the optical nonlinearities [4]. This presents a way to enhance entanglement in nonlinear systems, which are also of interest to us.

In recent years, driven nonlinear systems have attracted a great interest due to their applications in quantum-enhanced technologies and quantum information processing [8,9,10,11,12,13,14,15,16,17,18,19]. They are key ingredients in fields such as optomechanical systems [8,9,10], nanomechanical resonators [11,12,13,14,15,16,17], and superconducting quantum interference device (SQUID) [18,19], which are considered to be excellent candidates for quantum information [20,21,22]. Furthermore, in the studies related to driven nonlinear systems, the dependence of the system’s dynamic behavior on the driving frequency is of great interest [14,15,16,17,18,19]. However, as far as we know, the influences of the driving frequency on the entanglement in driven nonlinear systems have not been reported yet. Therefore, we perform an investigation to study the impact of driving frequency on the entanglement with two coupled driven nonlinear oscillators.

Specifically, the production of the entanglement entropy is explored in a coupled system, which is composed of two identical driven nonlinear oscillators. The system’s initial state is a product of coherent states and the entanglement between the subsystems is measured by the linear entropy. It is found that the entropy production varies significantly with the driving frequency. The dependence curve of the entropy production on the driving frequency has a remarkable peak, near which the entanglement between the subsystems is greatly increased. Further analyzes show that the entropy production is related to the energy levels, which can be influenced by the driving force. This leads to the dependence of the entanglement entropy on the driving frequency. The energy levels, participating in the quantum evolution, can be evaluated by the Fourier spectrum of the correlation function and the quantum uncertainty. It is showed that the entropy production increases with the density and width of the Fourier spectrum of the correlation function. Besides, good correspondence is observed between the frequency response curves of the quantum uncertainty and the entropy production. These results indicate that driving frequency influences entropy production by affecting the energy levels.

In the following sections, investigations are carried out on the dependence of the entanglement entropy production on the driving frequency with a bipartite driven nonlinear system. In Section 2, we introduce the system of interest, the linear entropy, and the initial state of the system. Section 3 presents the variance of the entropy production with the driving frequency. In Section 4, we analyze the relation of the entropy production and the external driving from the perspective of the quantum evolution. In the final section, we present our conclusions and ideas for future work.

## 2. System of Interest

The system is composed of two identical Duffing oscillators, which are driven by the periodical external source. Its Hamiltonian reads,

(1) $$H=\sum_{k=1}^{2}\left(\frac{p_{k}^{2}}{2m}+\frac{1}{2}\alpha m\omega_{0}^{2}q_{k}^{2}+\frac{1}{4}\beta q_{k}^{4}\right)+f\left(q_{1}+q_{2}\right)\sin\left(\omega t\right)+\gamma q_{1}q_{1},$$

where the last term on the right-hand side denotes the coupling between the subsystems and γ is the coupling strength. Physical systems such as Equation (1) include vibrating molecules [23], modes of the electromagnetic field [24,25,26], and coupled optomechanical systems [27,28]. The subsystem, i.e., the Duffing oscillator, is of both theoretical and experimental interest. It is a practical model of optomechanical and nanomechanical devices [14,15,16,17,18].

For system Equation (1), the entanglement between the subsystems can be measured by the linear entropy. The latter reads,

(2) $$S=1-Tr(\rho_{k}^{2}),$$

where $\rho_{k}$ is the reduced density of the *k*-th subsystems (k=1,2). The latter is an approximation of the quantum von Neumann entropy and is conventionally calculated in phase space. As the entanglement between the subsystems increases, the value of *S* increases from 0 to 1.

In the following sections, we focus our attentions on the dependence of the entanglement entropy *S* on the driving frequency ω. The parameters except ω are kept constant. Their values are α=1, β=0.1, γ=0.1, and f=0.5 in natural units (ℏ=1, m=1, $\omega_{0}=1$).

The initial state of the system is a direct product state of two coherent states, which correspond to the ground states of the Harmonic oscillators (the vacuum states). In the phase space, it reads $\langle q_{1},q_{2}|\Psi \rangle =N\exp\left[-\left(q_{1}^{2}+q_{2}^{2}\right)/\sigma^{2}\right]$, where *N* is the normalization constant and $\sigma^{2}=2$ in natural units. It can be regarded as a direct product of two Fock states. Both Fock and coherent states play important roles in quantum information and quantum optics [29].

## 3. Entanglement Entropy and Its Frequency Response

**Figure 1:**
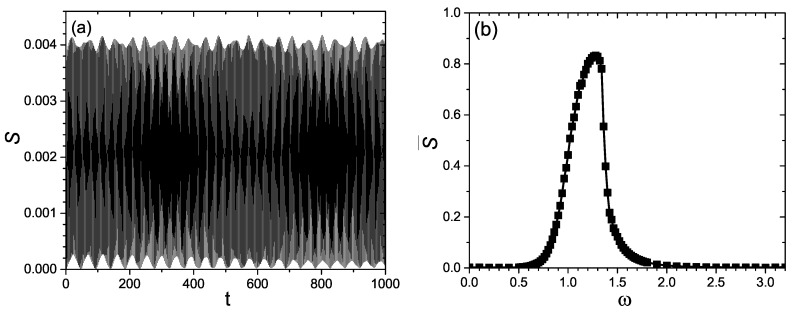
(**a**) Time evolution of the entropy *S* for ω=0. (**b**) Average of the entropy over time $\bar{S}$ versus the driving frequency ω (the increment of ω is 0.02 for ω∈[0.5,1.8] and 0.1 for the other intervals). When ω=0, the external driving is zero and the system is isolated.

The value of the entanglement entropy is equal to zero when t=0. It grows with the time during the quantum evolution because of the coupling between the subsystems. When ω=0, the system is isolated and not driven by the external force. The time evolution of the entanglement entropy for ω=0 is presented in Figure 1a. Figure 1a shows that the entropy oscillates beseem 0 and $4.2\times 10^{-2}$ during the time evolution. Its average over the time interval [0,1000] is $2.1\times 10^{-2}$, which is close to zero. Accordingly, the entanglement between the two subsystems is very small, when ω=0 and the system is unperturbed by the driving force. This is evidently unsatisfactory from the perspective of achieving entanglement. As mentioned above, entanglement is an essential resource in quantum information. It would be exciting if we could find simple ways to enhance the entanglement in the above system. As an attempt, we apply periodic external driving force to the system and explore whether the entanglement production can be influenced by tuning the driving frequency.

**Figure 2:**
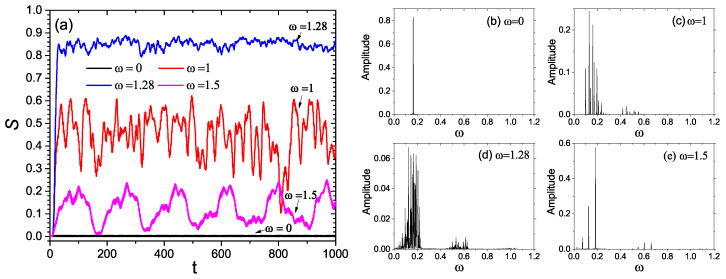
(**a**) Time evolution of the entropy *S* for ω=0 (black line), ω=1 (red line), ω=1.28 (blue line), and ω=1.5 (magenta line). Fourier spectra of the correlation function C(t) for (**b**) ω=0, (**c**) ω=1, (**d**) ω=1.28, and (**e**) ω=1.5.

Specifically, ω is increased from 0 to 3. In this case, the system is driven by the external driving force shown in Equation (1). The numerical results show that the entanglement entropy of the system can be influenced by the driving frequency ω. This can be seen from Figure 1b and Figure 2a. Figure 1b displays the variance of the entropy production with ω, whereas Figure 2a exhibits the time evolution of the entropy *S* for four typical driving frequencies. As illustrated by Figure 2a, the value of S(t) oscillates around some mean value after its initial growth. Therefore, in Figure 1b, the entropy production is measured by its average over time, i.e., $\bar{S}=\int_{0}^{\tau }S\left(t\right)dt$ (τ=1000).

From Figure 1b and Figure 2a, it is interesting to see that the entanglement entropy significantly depends on the the driving frequency. In Figure 1b, the value of $\bar{S}$ is very small when ω is smaller than 0.5. As ω increases from 0.5 to 1.28, the value of $\bar{S}$ increases dramatically and reaches its maximum near ω=1.28, after which it decreases rapidly to almost zero. Accordingly, the driving frequency response curve of $\bar{S}$ shows a sharp and intense peak around ω=1.28. Near the above peak, the entanglement production is greatly enhanced by the driving force. This can be further confirmed by Figure 2a.

Figure 2a shows the time evolution of the entropy *S* for four typical values of the driving frequency ω. In Figure 2a, the values of ω are 0 (black line), 1 (red line), 1.28 (blue line), and 1.5 (magenta line). From Figure 2b, one can further obverse the influences of the driving frequency ω on the production of the entanglement entropy *S*. In Figure 2b, after the initial increase, the entropy for ω=1.28 oscillates with time ~0.85. Both the growth rate and average value of the entropy *S* for ω=1.28 (the peak frequency in Figure 1b) is much faster than those for ω=0, ω=1, and ω=1.5.

The above observations suggest that the entanglement entropy between the two coupled driven nonlinear oscillators can be significantly influenced by the driving force. Near the peak of the curve of $\bar{S}\left(\omega \right)$, the value of the linear entropy is close to 0.9, and thus the entanglement between the subsystems is close to the maximal entanglement, as displayed by Figure 1b and Figure 2a. In sharp contrast, the entanglement entropy is almost zero in the absence of the external driving, as can be seen from Figure 1a,b. Accordingly, the entanglement can be greatly increased by tuning the driving frequency. This provides a way to enhance the entanglement via the driving frequency, as driven nonlinear systems are widely used in quantum technologies and quantum information processing.

## 4. Entropy Production during the Quantum Evolution

The influences of driving force on the entropy production can be understood from the evolution of the system. According to the methods of Liouville dynamics [30,31], the time evolution of the density ρ for a quantum system like Equation (1) follows,

(3) $$\rho^{w}\left(x,t\right)=\sum_{n,n^{\prime }}c_{n,n^{\prime }}^{w}\rho_{n,n^{\prime }}^{w}\left(x\right)\exp\left[-i\lambda_{n,n^{\prime }}t\right],$$

where $\rho^{w}\left(x,t\right)$ is the Wigner–Weyl transform of ρ and x denotes $\left\{q_{1},q_{2};p_{1},p_{2}\right\}$. $\rho_{n,n^{\prime }}^{w}$ are the eigenstates of L(x) ($L=\hbar^{-1}\left[H,\right]$). In other words, $\rho_{n,n^{\prime }}^{w}$ are the Wigner–Weyl transform of $|\varphi_{n}\rangle \langle \varphi_{n^{\prime }}|$, where $|\varphi_{n}\rangle$ ($|\varphi_{n^{\prime }}\rangle$) is the eigenstates of the system Equation (1) with *n* ($n^{\prime }$) being the quantum numbers. $\rho_{n,n^{\prime }}^{w}$ is time-independent and satisfies

(4) $$L\left(x\right)\rho_{n,n^{\prime }}^{w}\left(x\right)=\lambda_{n,n^{\prime }}\rho_{n,n^{\prime }}^{w}\left(x\right),$$

where $\lambda_{n,n^{\prime }}=\left(E_{n}-E_{n^{\prime }}\right)/\hbar$ with $E_{n}$ ($E_{n^{\prime }}$) being the eigenvalues corresponding to $|\varphi_{n}\rangle$ ($|\varphi_{n^{\prime }}\rangle$).

The linear entropy can be written as S=1−P, where $P=Tr_{2}\left(\rho_{1}^{2}\right)$ is the purity. By means of Equations (3) and (4), *P* satisfies

(5) $$\begin{array}{c} P=\int dq_{2}\left[\int dq_{1}\rho \left(x,t\right)\right]\left[\int d{q^{\prime }}_{1}\rho \left(x^{\prime },t\right)\right]\\ \approx \sum_{n,n^{\prime }}\sum_{k,k^{\prime }}e^{-\frac{i}{\hbar }\left(E_{n}-E_{n^{\prime }}+E_{k}-E_{k^{\prime }}\right)t}\int dq_{1}dq_{2}d{q^{\prime }}_{1}dp_{1}dp_{2}\rho_{n,n^{\prime }}^{w}\left(x\right)\rho_{k,k^{\prime }}^{w}\left(x^{\prime }\right), \end{array}$$

where $x^{\prime }$ indicates $\left\{q_{1}^{\prime },q_{2};p_{1},p_{2}\right\}$.

As shown by Equation (5), the time evolution of the entropy is dependent on the exponential function. The latter is determined by the energy levels $\left\{E_{n}\right\}$ involved in the quantum evolution. For a driven system like Equation (1), the presence of the driving force can induce transitions between energy levels. Therefore, it can influencing the time evolution of the entanglement entropy by affecting the energy levels participating the quantum evolution. This is similar to what happens in a classical driven system near the region of resonance [32,33]. In other words, the response of the entropy production to the driving frequency can be regarded as some kind of resonance in the entropy production. The driving frequency that corresponds to the peak of $\bar{S}\left(\omega \right)$ corresponds to the fundamental frequency of the system. The influences of the external driving on the quantum evolution and the entropy production are greatly strengthened, when the driving frequency approaches the fundamental frequency of the frequency response curve of the entropy production. This induces the emergence of the peak of $\bar{S}\left(\omega \right)$ (see Figure 1b). On the contrary, the influences of the driving on the quantum evolution and the dependence of the entropy production on the driving frequency are weak, as the driving frequency is away from the peak frequency. These can be confirmed by the following numerical investigations that are related to the correlation function.

The energy levels participating in the evolution of the quantum state can be investigated by the Fourier analysis of the correlation function C(t) with $C\left(t\right)=\langle \Psi (0)|\Psi (t)\rangle$, where $|\Psi (t)\rangle$ is the state of the system at the time *t*. It is easy to know $|\Psi (t)\rangle =\sum_{n}\epsilon_{n}\exp\left(-iE_{n}t/\hbar \right)|\varphi_{n}\rangle$ and $|\Psi (0)\rangle =\sum_{n}\epsilon_{n}|\varphi_{n}\rangle$. Thus, $C\left(t\right)=\sum_{n}{\left|\epsilon_{n}\right|}^{2}\exp\left(-iE_{n}t/\hbar \right)$. C(t) can be viewed as a time signal with frequencies $\left\{E_{n}/\hbar \right\}$. Therefore, the energy levels involved in the quantum evolution can be revealed by the Fourier spectrum of C(t) with respect to *t* [34]. According to the theory of Fourier analysis [34], the spectral lines in the Fourier spectrum of C(t) indicate the energy levels $\left\{E_{n}\right\}$ involved in the quantum evolution.

In Figure 2b–d, we present the Fourier spectra of C(t) for ω=0, ω=1 and ω=1.28 and ω=1.5. As is illustrated by Figure 2b–d, the Fourier spectrum significantly depends on the driving frequency. Comparing Figure 2a and Figure 2b–d, one can find that the entropy production increases with the density and width of the Fourier spectrum of C(t). This supports the above argument that the driving frequency influences the entropy production by means of affecting the energy levels and the influences of the driving force on the entropy production can be viewed as some kind of resonance.

**Figure 3:**
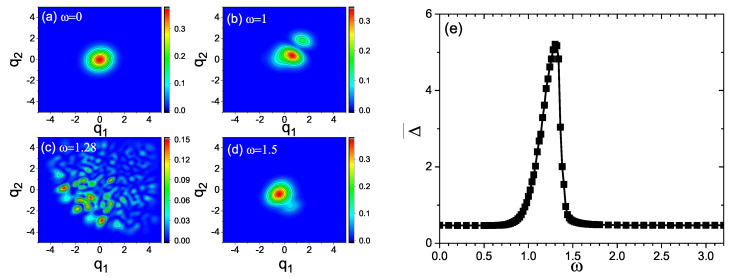
(**a**–**d**) Probability distribution $|\langle q_{1},q_{2}|\Psi (t)\rangle {|}^{2}$ for (**a**) ω=0, (**b**) ω=1, (**c**) ω=1.28 and (**d**) ω=1.5. (**e**) Average of the uncertainty over time $\bar{\Delta }$ versus the driving frequency ω (the increment of ω is 0.02 for ω∈[0.5,1.8] and 0.1 for the other intervals).

Increase of the energy levels can induce increase the interference between energy levels. The interference can be seen from the distributions of the probability amplitude in the phase space, i.e., ${\left|\langle q_{1},q_{2}|\Psi \left(t\right)\rangle \right|}^{2}$. The latter are displayed in Figure 3a–d. As can be found from Figure 3a–d, the interference fringes and dispersion of the quantum state for ω=1.28 are much more significant than those for ω=0, ω=1 and ω=1.5.

The dispersion of the quantum state can be evaluated by the uncertainty of the quantum state in position, i.e.,

(6) $$\Delta ={\langle {\langle q_{1}\rangle }^{2}-\langle q_{1}^{2}\rangle \rangle }^{1/2}{\langle {\langle q_{2}\rangle }^{2}-\langle q_{2}^{2}\rangle \rangle }^{1/2}.$$

The increase of quantum interference accompanies with the increase of quantum uncertainty Δ. In this case, Δ can be used as an indicator as the quantum interference and the energy levels participating the quantum evolution.

Similar to Figure 1b, $\bar{\Delta }=\int_{0}^{\tau }\Delta dt$ is used to estimated the quantum uncertainty Δ during the time evolution. The frequency response of $\bar{\Delta }$ is given in Figure 3e. From Figure 3e, we can clearly see the variation of $\bar{\Delta }$ with the driving frequency ω. Especially, a significant peak can be found in the curve of $\bar{\Delta }\left(\omega \right)$. Its emergence suggests that during the quantum evolution, the dispersion of the wave function and the energy level transitions are greatly stimulated by the driving force near the peak frequency. This confirms the above argument that the frequency of driving force influences the evolution of the wave function in a way analogous to resonance. Comparing Figure 1b and Figure 3e, good correspondence can be found between the frequency response curves of $\bar{S}\left(\omega \right)$ and $\bar{\Delta }\left(\omega \right)$. The peaks of the curves of $\bar{S}\left(\omega \right)$ and $\bar{\Delta }\left(\omega \right)$ in Figure 1a and Figure 3e occur around the same driving frequency. This further supports the above argument about the influences of the driving frequency on the entropy production during the quantum evolution.

## 5. Discussion

As mentioned above, entanglement is the core resource of quantum information, whereas driven nonlinear systems are widely used in quantum technology. The results presented in this work provide insights into the dependence of the entanglement entropy on the frequency of the external driving in two coupled driven Duffing oscillators. It is shown that the bipartite entanglement in driven nonlinear systems can be significantly increased through tuning the frequency of the external periodic driving. In this view, this work provides a convenient way to enhance the entanglement in driven nonlinear systems by mean of the external modulation (laser beam, electromagnetic field, etc.). Meanwhile, our investigations also sheds some light on role of the driven nonlinear systems in quantum information technologies. For instance, the quantum correlations beyond entanglement may be modulated by the external driving in driven nonlinear systems, since one of the attractive features of driven nonlinear systems is that it can be controlled and modulated by the external driving. Even for undriven nonlinear quantum systems, external drive (e.g., external field or electric source) can be thought of to be applied to influence the entanglement between the subsystems.

In physical realization, a quantum system is open and influenced to some extent by the surrounding environment [4,35,36,37,38,39]. During recent years, the loss of quantum correlations due to the environment has received considerable attention [39,40,41]. In this view, it is necessary to further explore the relation of the entanglement dynamics to the driving frequency in open systems. In view of the influences of driving force on the quantum evolution, it is believable that the enhancement of the entanglement by the driving frequency would be observed to some degree in the presence of the environment. Furthermore, in driven nonlinear systems, the influences of the environment on the entanglement (e.g., the loss of quantum correlation) may be decreased by tuning the driving frequency, as the entanglement is found to be enhanced by the driving frequency in this work. These need to be studied in future researches.

As mentioned above, this work is focused on the dependence of the entanglement on the driving frequency. It needs to be pointed out that all the parameters in Hamiltonian Equation (1) can affect the time evolution of the entanglement entropy. For different parameter values (e.g., different values of α and β), the shape and position of the response curve of the entropy production will be different. However, it ensures the frequency response curve of the entanglement entropy could be observed for different parameter values, because of the influences of the driving frequency on the evolution of the wave function. The roles of the other parameters except the driving frequency in the quantum evolution and the entropy production will be clarified in the forthcoming studies. Furthermore, as illustrated by Equation (1), the system is composed of two Duffing oscillators. The subsystem’s potential, i.e., $V\left(q_{k}\right)=\alpha \omega_{0}^{2}q_{k}^{2}/\left(2m\right)+\beta q_{k}^{4}/4$ (k=1,2), contains both quadratic and cubic terms. It is dependent on the parameters α, *m*, and β. Accordingly, the behavior of the vacuum of the system also depends on the parameters of the potential (i.e., α, *m*, and β). Multiple equivalent vacuums may exist for some combination of the parameters of the subsystem’s potential [42,43,44,45,46,47,48]. This, in particle physics, is closely related to spontaneous symmetry breaking or possible phase transitions [43,44,45,46,47,48,49]. The vacuums of the system Equation (1) for different parameter values need to be explored in the future.
